# Nanoplasmonic Strip Test for Salivary Glucose Monitoring

**DOI:** 10.3390/nano12010105

**Published:** 2021-12-29

**Authors:** Helena Torné-Morató, Paolo Donati, Pier Paolo Pompa

**Affiliations:** 1 Nanobiointeractions & Nanodiagnostics, Istituto Italiano di Tecnologia (IIT), via Morego, 30-16163 Genova, Italy; helena.tornemorato@iit.it (H.T.-M.); paolo.donati@iit.it (P.D.); 2 Department of Chemistry and Industrial Chemistry, University of Genova, via Dodecaneso, 31-16146 Genova, Italy

**Keywords:** colorimetric sensor, gold nanoparticles, plasmonics, hyperglycemia, point-of-care

## Abstract

Nowadays, there is an increasing interest in Point-of-care (POC) devices for the noninvasive glucose assessment. Despite the recent progress in glucose self-monitoring, commercially available devices still use invasive samples such as blood or interstitial fluids, and they are not equipment-free and affordable for the whole population. Here, we report a fully integrated strip test for the semi-quantitative detection of glucose in whole saliva. The colorimetric mechanism consists of an enzyme-mediated reshaping of multibranched gold nanoparticles (MGNPs) into nanospheres with an associated plasmonic shift and consequent blue-to-red color change, clearly detectable in less than 10 min.

## 1. Introduction

Diabetes mellitus is a worldwide prevalent disease. In 2019, it was estimated that 463 million adults suffer from it, and the incidence rate will continue to increase in the coming years [1]. Incorrect drug administration is especially dangerous and can lead to hyperglycemic or hypoglycemic states, which represent an immediate life threat and an increase in health complications in the long term. Hence, glycaemia must be strictly regulated by frequent glucose self-testing to maintain it in a homeostatic range [2]. Compliant blood glucose monitoring is also interesting for a variety of nondiabetic subjects, from professional athletes chasing to improve their performances [3,4] to elderly Parkinson’s disease patients with glucose fluctuations [5].

In this framework, point-of-care (POC) sensors are a key tool to fulfill the analytical demand. The POC aim is to adapt the prognosis to the patients’ vicinity and improve their quality of life. REASSURED [6] criteria sum up the fundamental features that these devices must have to be considered in POC. Currently, there is a variety of commercial glucose detection options based on different mechanisms [7,8]. However, these devices do not fully meet the POC requirements, mainly because they do not facilitate the specimen collection, due to the use of invasive samples. Moreover, they are not equipment-free and are not affordable for low/middle-income countries [8,9,10]. Among the commercial tools, glucometers are widely spread, thanks to their sensitivity and reliability. Additionally, implantable devices capable of continuous glucose monitoring are rising due to a more accurate glycaemia control compared to punctual measurements. Nonetheless, these devices use invasive samples like capillary blood obtained by a finger-prick or interstitial fluids. These techniques may cause pain and secondary complications, such as lichenification, eczema, wounds and subsequent infections [11], which increase the overall treatment cost [12,13,14].

Noninvasive fluids like urine, saliva, sweat, and tears represent a promising alternative for the glycaemia measurement. Urine, sweat, and tears show high glucose concentration and remarkable correlation with blood levels; however, they require collection protocols that may generate discomfort or are not applicable to all patients. In case of sweat, it includes exercise or high temperature exposure, while tears require induced lacrimation or the use of an extra microfluidic collection device [15,16]. Additionally, urine diagnostic value is limited, since the glucose concentration in urine may be variable and is not representative of a certain instant but is the average of the production/accumulation period, which can be several hours. Saliva is readily available and easy to collect at any time thanks to its physiological production rate of ca. 0.3 mL/min. Moreover, it displays a good glucose correlation with blood levels in ca. 1:60 ratio (saliva: 1.5–2.5 mg/dL and blood: 80–120 mg/dL) [17]. For these reasons, salivary secretions are a noteworthy candidate for noninvasive and sensitive glycaemia monitoring. On the other side, saliva presents a complex matrix and a significant inter- and intra-individual composition variation [18]. To overcome these challenges, some methods include a sample pre-treatment, which however may complicate the overall test usability [19,20].

In recent years, fully-integrated paper-based detection devices [21], like lateral flow assays (LFAs) [22], have become a popular type of sensing platforms. The use of paper and fibers (polymer- or glass-fiber) led to portable and cost-effective tools, in which these materials provide capillary sample flow for instrument-free reagent mixing. Solid supports can also store dried reagents to build a fully integrated sensing tool. Moreover, dried biological components and nanoparticles show an increased stability. All these features can be properly combined to create a device for automated glucose detection.

Herein, we report a fully-integrated POC device for the instrument-free naked-eye semi-quantitative evaluation of glucose in whole saliva. This sensor consists of a paper-based strip test, in which the optimized design and selected materials allow an automated colorimetric test. The detection mechanism has been developed optimizing a nanoplasmonic colorimetric method [23]. The prototype readout exhibits a clear color change from blue to red in only 10 min.

## 2. Results and Discussion

**Figure 1:**
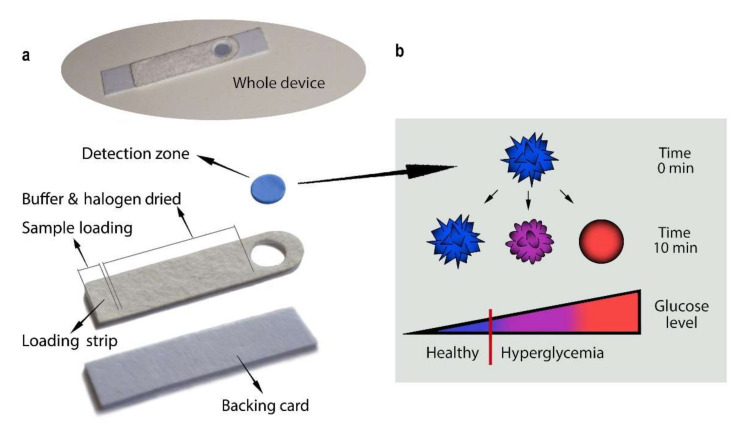
Device structural and functional scheme. (**a**) Representative photographs of the whole device and its comprising parts. The detection zone is constituted by a polyamide membrane functionalized with MGNPs and GOx. The loading strip is a Fusion5^®^ membrane, where the phosphate buffer and sodium iodide have been dried. The reaction zone and loading strip are held together on an adhesive backing card. (**b**) Sensing scheme and colorimetric results. This device is designed to recognize a healthy level of glucose and two increasing levels of hyperglycemia. Depending on the glucose concentration in the salivary fluid, MGNPs retain their surface structure (blue color), are partially (purple) or are totally (red) and reshaped into spherical GNPs. The colorimetric results can be read 10 min after the sample loading.

The reported strip test is constituted by a detection zone placed at the edge of a sample loading strip that also acts as a chemical reservoir (Figure 1). The sample loading strip is composed of a Fusion5^®^ membrane where phosphate buffer and sodium iodide have been dried. Fusion5^®^ membrane was chosen among different materials used with blood and saliva (MF1, LF1, GF/DVA, VF2) [24]. The cell separation capacity of Fusion5^®^ (pore size 2.3 μm) helps in filtering a complex matrix like salivary fluids. Iodine has been chosen for its higher activity compared to bromide or chloride [23] as a reaction booster, catalyzing the hydrogen peroxide reduction reaction. The detection zone consists of a polyamide membrane functionalized with multibranched gold nanoparticles (MGNPs) [25] (see Figures S1–S3 for their characterization) and Glucose Oxidase (GOx). GOx enzyme is part of the sensing mechanism, but it also creates a protecting layer for MGNPs, increasing the platform storage stability. The device has been optimized in terms of both geometry and materials. Polyamide membrane shows an ordered structure that allows a fine particle deposition, resulting in a vivid color, compared to cellulose and its derivatives (Figures S4 and S5a,b). The structural design of the strip has been developed to ensure optimal and uniform sample diffusion in the detection zone. Two flows take place inside and on top of the Fusion5^®^ glass fiber, delivering the necessary quantity of the sample mixed with the right amount of the halogen. The loading strip was design to surround the polyamide, creating a well-like region where the reaction mix (sample and halogen) accumulates on top of the detection zone (see Figure 1a).

The sensing mechanism is based on the plasmonic shift of MGNPs when reshaped into spheres by an enzyme-mediated reaction [26]. As a first step, GOx catalyze salivary glucose oxidation producing gluconic acid and hydrogen peroxide, which in turn reacts with the re-suspended iodine ion creating di-iodine as an intermediate species:

(1) $$H_{2}O_{2}+2I^{-}+2H^{+}\to I_{2}+2H_{2}O$$

Di-iodine further interacts with other iodine ions generating tri-iodide:

(2) $$I_{2}+I^{-}\to I_{3}^{-}$$

Tri-iodide is able to oxidize gold atoms on the MGNP tips [27,28]:

(3) $$2Au+I_{3}^{-}+I^{-}\to 2AuI_{2}^{-}$$

The gold atoms oxidation and reduction reactions cause their migration from tips to lower energy position (core surface), reshaping the gold nanoparticles into spheres that are more thermodynamically stable [23]. This reaction would transform MGNPs (LSPR ʎ_max_ = 650 nm) into spheres (LSPR ʎ_max_ = 550 nm). Tuning this reaction, the device was set up to discriminate three levels of glucose in saliva: a healthy level and two increasing levels of hyperglycemia. In normal glycemic states, the color of the detection zone remains blue, while in hyperglycemic states the color first shifts to purple and then to red (Figure 1b).

**Figure 2:**
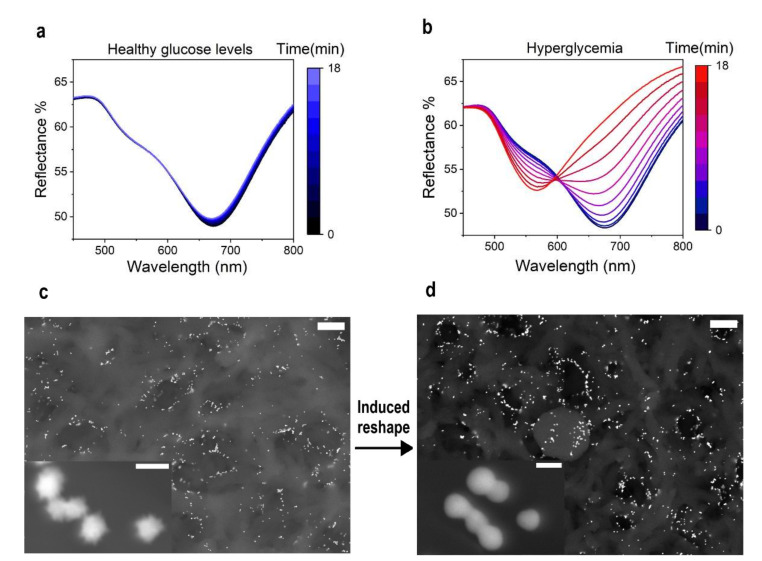
Detection mechanism characterization. Reflectance spectroscopy (diffuse mode) spectra of (**a**) a healthy glucose level saliva sample (1.5 mg/dL) and (**b**) a sample spiked with D-(+)-Glucose to simulate a hyperglycemic state (9.5 mg/dL), monitored for 18 min. The sample with healthy levels of glucose maintained its reflectance spectra stable over the time, while the hyperglycemic sample reflectance peak shifted from 670 to 560 nm. Bottom: HR-SEM micrographs of polyamide membranes casted with homogenously dispersed MGNPs before the test (**c**) and reshaped GNP spheres after the test (**d**) (scale bars: low magnification 1 µm; high magnification 50 nm).

Reflectance spectroscopy was used to characterize the target-induced color change. Non-treated saliva (1.5 mg/dL) and samples spiked with a glucose standard solution to simulate a strong hyperglycemic state (9.5 mg/dL) were used to record the diffuse mode reflectance spectra (see Figure S6) of the detection zone for 18 min (Figure 2a,b). The initial spectra of all samples displayed a minimum reflectance peak at 670 nm, while most of the reflected light was in the range from 450 to 500 nm. The spectral profile of the sample with a basal glucose concentration (1.5 mg/dL) did not manifest significant changes after 18 min. However, in case of hyperglycemia, the minimum reflectance shifted to a wavelength of 560 nm and the reflected percentage increased in the range from 650 to 800 nm. All initial samples were blue in terms of perceived color, while only the spiked one turned red during the assay, as showed by the spectral shift (Figure 2a,b). The reshaping process that occurs in the detection zone was also characterized using high resolution scanning electron microscopy (HR-SEM). HR-SEM micrographs display a sensor area before (Figure 2c) (see also Figure S5c,e) and after testing with a hyperglycemic sample (Figure 2d) (see also Figure S5d,f). The polyamide membrane substrate, used as the detection zone, presented a monodispersed and stable layer of active MGNPs (pre-test) and spherical GNPs (post-test). Thanks to this solid support, we could increase the particle stability avoiding aggregation processes that can interfere, affecting the assay quality. In higher magnification images (insets of Figure 2c,d), multibranched and spherical structures could be appreciated before and after the test, respectively, confirming that the color change was primary due to the reshaping mechanism.

Detailed list of chemicals, instruments, and procedures are fully described in the Supplementary Materials.

**Figure 3:**
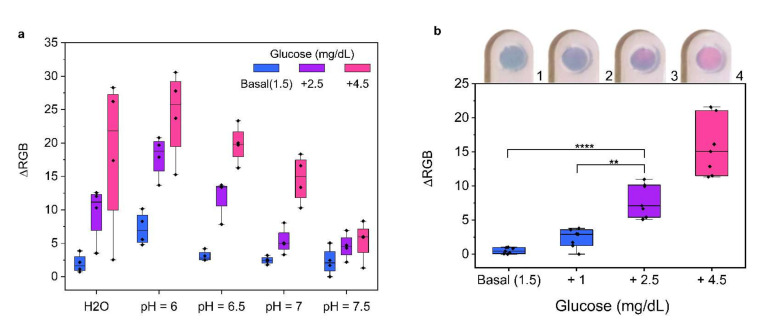
Device optimization and performance. (**a**) Buffering and pH condition optimization of the detection zone using four healthy donors’ saliva samples. The non-buffered condition (H_2_O) was prepared with deionized water, while buffered conditions were prepared with phosphate buffer (300 mM) at pH 6, 6.5, 7, 7.5. Considering a basal glucose concentration of 1.5 mg/dL, samples were spiked with 2.5 and 4.5 mg/dL. (**b**) Device performance was assessed with seven saliva samples from healthy volunteers. Samples were spiked with 1, 2.5 and 4.5 mg/dL. One-way ANOVA and Tukey’s multiple comparison test were used to determinate the statistical significance (**** *p* < 0.0001 and ** *p* < 0.01). The insert image is representative of the assay results of four different glucose concentrations (1, 2, 3 and 4 correspond to basal glucose concentration 1.5 mg/dL and spikes of 1, 2.5, 4.5 mg/dL, respectively). The RGB coordinates values were always measured from pictures at t = 0 and t = 10 min with ImageJ software. In both experiments, all conditions were tested in triplicates.

Environmental pH strongly affects the kinetics of the MGNP reshaping process. In particular, acid conditions accelerate the etching of the particles [23]. Since salivary pH ranges from 5.8 to 7.5 [29], sample buffering was a key factor to ensure the test reproducibility across donors. As shown in Figure 3a, tests conducted directly on the detection zone (see also Figure S7) confirmed that non-buffered samples from different donors showed low reproducibility. On the contrary, pH-controlled conditions allowed a better reproducibility in terms of ΔRGB. Specifically, pH 6.5 exhibited the best discrimination between basal and unhealthy glucose levels. In addition, the basal glucose condition exhibited a stable color without any detectable shifting towards the red.

To study the device performances, saliva samples from healthy donors were tested. Considering a healthy population, the basal salivary glucose level is on average ca. 1.5–2.5 mg/dL (83.3 µM) [17], hence we considered this value as representative of a healthy state. Samples were spiked with a glucose standard solution up to 2.5, 4, 6 mg/dL. After 10 min (see Figure S8), the color change was evaluated by RGB analysis on photos and with a smartphone app. An illustrative video of the device color change is available (see video S1). The data showed a remarkable correlation (R^2^ = 0.94) between the color change, expressed in terms of ΔRGB, and the glucose concentration (Figure S9). The device performances were tested on saliva samples from seven volunteers: simulated hyperglycemic conditions (4 and 6 mg/dL), which represent a health threat, showed a significant color change, while basal (1.5 and 2.5 mg/dL) conditions maintained a stable blue color. Tuning the chemical conditions, a strong color difference between healthy and hyperglycemic conditions could be achieved. Figure 3b reveals a significant difference in ΔRGB with clearly visible color change (inset Figure 3b), useful for instrument-free applications. Importantly, this integrated device was developed to host a buffering step, a filtering process, and the stabilization of the nanoparticles in the detection zone. The combination of such features allowed us to avoid any additional saliva pre-treatment or purification steps, which are currently required for commercially available POC sensors.

Overcoming the challenge of the high variability of saliva composition, the optimized test strip was capable to discriminate between two health threatening hyperglycemic states (4 and 6 mg/dL) and a healthy condition (1.5 mg/dL) with a statistical significance of *p* < 0.0001. This device was tuned to improve the naked-eye evaluation, thereby it achieves the best color difference between +1 and +2.5 mg/dL. Such conditions lead to a limit of detection (LOD) of 2.0 mg/dL. In case of smartphone-based analysis, the sensor’s LOD can be stressed up to 0.4 mg/dL [23]. Our device also has a fast response time, since the results could be read 10 min after the sample loading, useful to manage properly hyperglycemic states.

## 3. Conclusions

We succeeded in the optimization of a fully integrated POC strip test for the semi-quantitative detection of glucose in whole saliva. The remarkable sensitivity of the test allows the discrimination of a healthy state from initial hyperglycemia (4 mg/dL). The use of a noninvasive fluid represents an improvement for the operator’s well-being, increasing the overall process comfort and avoiding secondary health complications for high frequency users. Moreover, the sensing mechanism is reproducible and specific, without the need of a saliva filtration or pre-treatment steps. The fully-integrated design of the device reduces the user’s operational steps to sample loading only, without specific training or extra instrumentation. All these features make this device a fit candidate to cover the current need for a noninvasive method of glucose self-testing.

## Data Availability

The data of this study are available from the corresponding author upon reasonable request.

## Supplementary Materials

The following are available online at https://www.mdpi.com/article/10.3390/nano12010105/s1, Figure S1: Dynamic Light Scattering (DLS) analysis, Figure S2: Transmission Electron Microscopy (TEM) analysis, Figure S3: UV-visible spectroscopy analysis, Figure S4: Substrate material screening and deposition optimization, Figure S5: Scanning electron microscopy (SEM) analysis, Figure S6: Reflectance spectra analysis, Figure S7: Buffering pH condition optimization, Figure S8: Device performance dependence over time, Figure S9: Analytical curve used to assess LOD and LOQ of the device. Video S1: Representative video of the device.
