# Supplementary material for: Nanoplasmonic Strip Test for Salivary Glucose Monitoring
Source: Nanomaterials (Basel). 2021 Dec 29;12(1):105. doi: 10.3390/nano12010105 (PMC8746375; doi:10.3390/nano12010105)
Supplement: Supplementary file 1 [file nanomaterials-12-00105-s001.zip › nanomaterials-1530466-supplementary.pdf]

nanomaterials

Supplementary material

Nanoplasmonic strip test for salivary glucose monitoring

Helena Torné-Morató [1,2], Paolo Donati [1] and Pier Paolo Pompa [1*]

[1]Nanobiointeractions&Nanodiagnostics, Istituto Italiano di Tecnologia (IIT), Via Morego, 30 – 16163 Genova (Italy); helena.tornemorato@iit.it (H.T.M.); paolo.donati@iit.it (P.D.)
[2]Department of Chemistry and Industrial Chemistry, University of Genova, Via Dodecaneso, 31 – 16146 Genova, Italy; helena.tornemorato@iit.it (H.T.M.)
* Correspondence: pierpaolo.pompa@iit.it (P.P.P.)

1. Materials and Methods

Reagents: Hydrogen Tetrachloroaurate ($HAuCl_4$*XH_2O, Au 49% minimum, 42803) and Hydroxylamine sulfate (H_3NO•$0.5H_2SO_4$, 88944) were purchased from Alfa Aesar (Kandel, Germany). Potassium Phosphate monobasic (KH_2PO_4, P5655), 2-[4-(2-hydroxyethyl) piperazin-1-yl] ethanesulfonic acid (HEPES) (BioUltra, for molecular biology, ≥99.5%, 54457), D-(+)-Glucose (C_6H12O_6 ACS reagent, G5767) and Glucose Oxidase from *Aspergillus niger* (Type X-S, lyophilized powder, G7141) were purchased from Sigma-Aldrich (Milano, Italy). Syringe filter holder (re-usable polycarbonate, 13 mm Ø, 0.5 cm^2 filtration area, 16514), polyamide membrane filters (pore size 0.2 µm, 25007-142------N) and cellulose acetate filter (0.2 µm, 11107--47------N) were purchased from Sartorius (Göttingen, Germany). Plastic cover (MicroAmp™ Optical Adhesive Film, 4311971), Potassium phosphate dibasic anhydrous (K_2HPO_4, Honeywell Fluka®, 60353) and Sodium iodine (NaI, Honeywell Fluka®, 03129) were purchased from Thermo Fisher Scientific (Rodano, Italy). Fusion 5® membrane (Whatman™, 8151-9915) and Nitrocellulose blotting membrane (pore size 0.2 µm, 10600001) were purchased from Cytiva (Cornella de Llobregat, Spain). Biopsy puncher (diameter 3 mm, Uni-core®, WB100039) were purchased from Whatman (Pero, Italy). Backing card (polyether, KN-P51060.18) were purchased from Kenosha (Amstelveen, The Netherlands). Needles (0.5 mL, Omnican 20®, 9161619S) were purchased from B. Braun (Milano, Italia). Polyvinylidene difluoride membranes (0.22 µm, Durapore®, GVHP01300) and D-(+)-Glucose standard solution (100g/L in H2O, Sigma-Aldrich®, G8644) were purchased from Merk (Roma, Italy). Cellulose nitrate membrane filters (0.45 µm, Whatman™, 7184-001) and filter paper (grade 1, Whatman™, 1001-090) were purchased from GE Healthcare Life Science (Milano, Italy).

Instruments: UV-vis spectra (200-800 nm) of the nanoparticle suspension were acquired by Thermo Fisher NanoDrop (Wilmington, United States of America) (wavelength Accuracy ± 1 nm, absorbance accuracy 3% at 0.74 Abs@350nm) equipped with a small volume cuvette. The transmission electron microscopy (TEM) images were acquired using a JEOL JEM 1400 microscope from JEOL (Akishima, Japan). Nanoparticle hydrodynamic diameter (Dh) was measured by Zetasizer Nano Range from Malvern Panalytical (Malvern, United Kingdom). The scanning electron microscopy (SEM) analysis was performed using a JSM-6490LA microscope from JEOL (Akishima, Japan). The reflectance spectra assay was conducted using a Cary Universal Measurement Accessory (UMA) accessory fitted in a Cary 4000 spectrophotometer from Agilent (Santa Clara, USA). The running strips were cut with a Plotter Laser CO_2 Desktop from WorkLineStore (Cuneo, Italy). Ultrapure deionized water was obtained from an Aurium mini–Ultrapure Water System from Sartorius (Göttingen, Germany). The Smartphone used to acquire images and test the free Colorgrab® app is a Xiaomi Redmi Note 7 with software MIUI 12.5.1 from Xiaomi (Beijing, China).

Saliva sample collection: Unstimulated saliva samples were donated by healthy volunteers following a specific protocol that consider possible interferences like food inges-

tion and oral hygiene. Hence, saliva collection was carried out 45 minutes after food ingestion or oral hygiene, and donors rinsed their mouth with water 10 minutes before the saliva collection. Sample were collected randomized at different moments of the day. Moreover, to avoid saliva stimulation, donors were not speaking or swallowing during the whole collection procedure. Samples were used immediately after their collection to simulate the use of this sensor in a real situation. The usage of saliva samples in this study was approved by the Ethical Committee of Regione Liguria (405/2020 – DB id 10787). All subjects gave written informed consent for the inclusion in the study.

Nanoparticle synthesis: Multibranched gold nanoparticles (MGNPs) were synthetized using a mediated seed growth procedure. Firstly, Citrate-capped gold nanoparticles (GNPs) were synthesized using Turkevich-Frens method[1,2]. Subsequently, MGNPs were grown employing an optimization of Maiorano-Pompa method[3]. The following reagents were added to a 25 mL of double distilled water under stirring: 1.5 mL of HEPES solution (1 M, pH = 7.0), 0.65 mL of sphere GNPs (1.9 nM), 0.075 mL of hydroxylamine sulfate (0.1M) and 0.036 mL of hydrogen tetrachloroaurate (0.1 M). For 10-15 minutes, the solution progressively shifts from red to a blue color. Residual reagents were washed from MGNPs by centrifugation (2600 RCF, 25 min). The characterization of the synthetized particles was performed with Dynamic Light Scattering (DLS), transmission electron microscopy (TEM) and UV-vis spectrophotometry.

Dynamic Light Scattering (DLS) analysis: MGNPs and sphere GNPs hydrodynamic diameter (Dh) (Figure S2a, b) was evaluated 3 times (each measurement is constituted by the sum of 10 runs) employing a Zetasizer Nano Range (Malvern Panalytical). Figure S1 values are an averaged of these measurements expressed as a frequency distribution of intensity, size, and volume (Figure S1 a, b, inserts).

Transmission Electron Microscopy (TEM) analysis: Sample preparation was conducted by drop casting 3 µL of MGNPs and sphere GNPs on separated grids (CF150-Cu-50 - Carbon Film 150 Mesh, Cu, 50/bx), posteriorly they were dried overnight using a vacuum chamber. The images in Figure S2a-c show sphere GNPs while Figure S2d-f are related to MGNPs, both set of images were acquired employing a JEOL JEM 1400 microscope

UV-visible spectroscopy analysis: Figure S4a correspond to a UV-vis spectra (400-800 nm) were obtained from 100 µL suspension of MGNPs, while Figure S3b correspond to a UV-vis spectra (400-700 nm) of sphere GNPs. These samples were assessed using a Thermo Fisher NanoDrop and a small volume cuvette as a loading tool.

Substrate screening: Six materials have been tested as a substrate to be functionalize with MGNPs. Circle pieces (6 mm Ø) of polyamide membranes, cellulose filter paper, cellulose acetate membrane, cellulose nitrate membrane, nitrocellulose membrane and polyvinylidene difluoride were drop casted with 0.4 µm of MGNPs (612.2 pM, λ_{max} = 647 nm, absorbance = 12, ε = 1.96 × 10^{10} $M^{-1}cm^{-1}$) and subsequently dried in a vacuum chamber. Polyamide membrane was chosen as the most suitable material, because when functionalized with nanoparticles it displays a higher color intensity (Figure S4a).

Detection zone preparation: The device detection zone is constituted by a polyamide membrane functionalized with MGNPs and Glucose oxidase (GOx). Firstly 0.2 µm porous polyamide filter membranes were placed on top of a syringe filter holder on a Kitasato becker connected to a vacuum pump. The polyamide membranes were initially washed with double distilled water, then 0.6 mL of a MGNPs suspension (28.5 pM, λ_{max} = 647 nm, absorbance = 0.9, ε = 31.13 × 10^9 $M^{-1}cm^{-1}$, in HEPES buffer 25 mM, pH = 7.0) were added to obtain a uniform layer of MGNPs on top of the membrane and subsequently re-washed with double distilled water. Then, the membrane was removed from the vacuum system and a 100 µL of GOx solution (1.5 mg/mL, 252 U/mL) were drop casted on the membrane and re-absorb the excess. Immediately, the membranes were dried under vacuum and stored in low humidity conditions (Figure S4b).

Device assembling: The Fusion5® running strips were cut with a laser to create the optimized strips shape of 5 x 20 mm with a 3.25 mm diameter hole in a strip end (Figure

1a). Subsequently, these strips were drop casted with 20 μL of potassium phosphate buffer (100 mM, pH = 6.5) and NaI (20 μM), then they were dried in a vacuum chamber for 2 hours. The functionalized polyamide membrane was cut in circular pieces of 3 mm of diameter with a biopsy puncher. The running strip and the polyamide membranes were attached to a backing card to obtain the final device.

Scanning electron microscopy (SEM) analysis: Samples for SEM analysis were prepared cutting circular piece (6 mm Ø) of polyamide membrane and two fully casted functionalized membrane: one before the assay and another at the endpoint of the assay. Field Emission Gun – Scanning Electron Microscope (FEG-SEM): The samples were characterized by HRSEM using a JEOL JSM-7500LA (JEOL, Tokyo, Japan) equipped with a cold field-emission gun (FEG), operating at 15 kV acceleration voltage. The sample was previously coated with carbon for increasing the conductivity. Back-scattered electrons were used to enhance the difference between the membrane and the AuNPs. Images in Figure S5a, b correspond to polyamide membrane filters (pore size 0.2 μm), (Figure S5 c, d, g) display MGNPs on polyamide membranes pre-test and (Figure S5 e, f, h) show sphere GNPs on polyamide membranes after test glucose spiked saliva.

Reflectance spectra analysis: Whole functionalized polyamide membranes were loaded with 900 μL of sample mix constituted by 600 μL of whole saliva and 300 μL of NaI (25 μM) solubilized in phosphate buffer (300 mM). Once the membrane was completely wet with the sample, the excess was reabsorbed and immediately covered with an optical plastic. The color change of a membrane treated with healthy donor saliva (Figure 2a), and another treated with 9.5 mg/dL D-(+)-Glucose spiked saliva (Figure 2b) was recorded using diffuse mode during 18 min. Figure S6 compare a reflectance spectroscopy in diffuse and spectral modes of the color change of a membrane treated with healthy donor saliva at an initial time.

Buffering pH condition optimization: The effect of buffering the saliva and the optimum pH was test using the device detection zone only, constituted by a polyamide membrane functionalized with MGNPs and GOx. NaI was dissolved in double distilled water (non-buffered) and sodium phosphate buffer (300 mM) at four pH: 6, 6.5, 7 and 7.5. Considering saliva basal glucose level of 1.5 mg/dL, saliva samples were spiked with 2.5 and 4.5 mg/dL of D-(+)-Glucose to simulate levels of 4, and 6 mg/dL glucose in saliva. Saliva samples were mixed with the NaI buffered solution in a 2:1 ratio and subsequently load on the functionalized polyamide membranes. This experiment was performed using samples provided by four saliva donors and each condition was tested in triplicates. The glucose-dependent assay's color change was estimated after 10 min (Figure 3a). Figure S7 exemplify the assay of all buffering and glucose conditions at time 0 minutes (Figure S7a) and the final color change at 10 minutes (Figure S7b) of one donor.

Device testing: To assess the device performance, seven saliva samples were collected from healthy donors and spiked to simulate pathological hyperglycemic conditions. Considering saliva basal glucose level of 1.5 mg/dL, saliva samples were spiked with 1, 2.5 and 4.5 mg/dL of D-(+)-Glucose to simulate different levels of 2.5, 4, and 6 mg/dL glucose in saliva. For this experiment, seven volunteers donated saliva samples and each glucose level was tested in triplicate. Subsequently, 45 μL of samples were load on the prototype devices and the glucose-dependent assay's color change was estimated at 5, 10 and 15 minutes. As displayed in Figure S8 the device resolution depends on assessing time. Time 10 minutes was selected as the optimum time to read the sensor for its better discrimination between glucose levels (Figure 3b). To better illustrate the color change of the device within 10 minutes, we included a video of four devices with non-spiked saliva and saliva spiked with 1, 2.5, and 4.5 mg/dL. The values obtained with different glucose concentration at 10 minutes were also employed as a calibration curve. This calibration curve was used to calculate the limit of detection (LOD) and limit of quantification (LOQ) with the following equations (Figure S9):

$$LOD = \frac{\left((y_{(blank)} + 3.3 * SD_{(blank)}) - a\right)}{b} \quad LOQ = \frac{\left((y_{(blank)} + 10.1 * SD_{(blank)}) - a\right)}{b}$$

Where: $Y_{(blank)}$ = blank mean, $SD_{(blank)}$ = standard deviation of the blank, a = intercept at the x axis and b = slope of the calibration curve. Since saliva contain glucose in a basal state is not possible to have a blank constituted by saliva without glucose. Hence, we use the basal saliva color change at 5 minutes as a blank.

Data collection and analysis: The data collection for the glucose assay, the optimization of buffering and pH effect was performed taking pictures at the initial and final time of the reaction with a smartphone camera. To standardize light conditions, the samples were placed in an internal black matte opaque box and using the smartphone torch as a unique source of illumination. The smartphone camera parameters were fixed at (ISO = 100 and speed = 1/1000 sec) to avoid possible fluctuations. The subsequent collection of the RGB coordinates was conducted at the time of the assays with Colorgrab® (smartphone app) and posteriorly with ImageJ® program from images taken with the smartphone. The color change was evaluated calculating the final RGB coordinates difference from the initial using the following formula:

$$\Delta RGB = \sqrt{(R_{t1} - R_{t0})^2 + (G_{t1} - G_{t0})^2 + (B_{t1} - B_{t0})^2}.$$

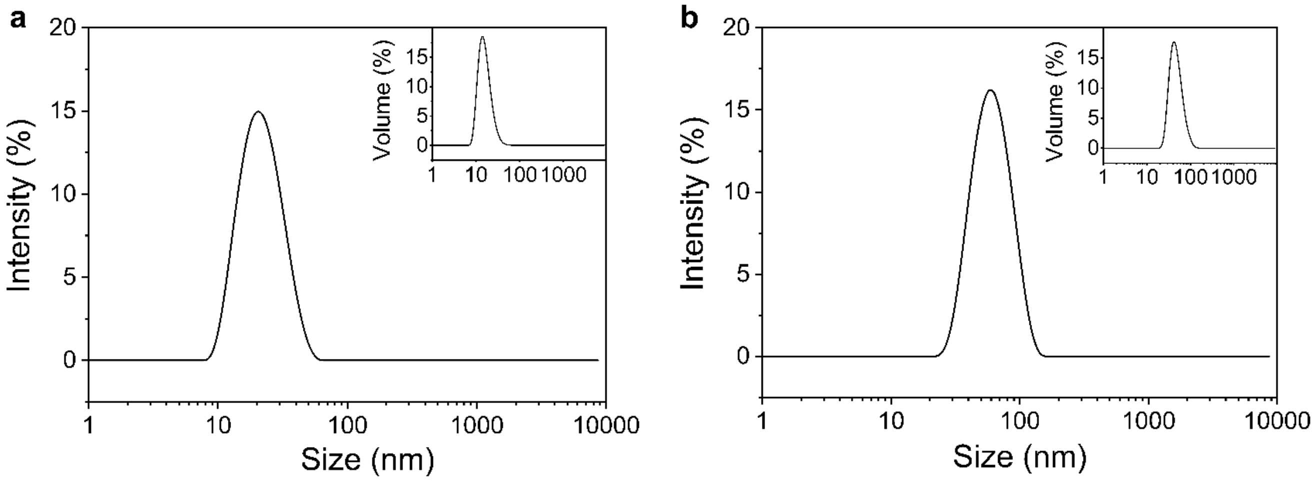

Figure S1. DLS measurements of (**a**) citrate-capped gold nanoparticles (GNPs) used as seeds (Dh = 21 nm) and (**b**) MGNPs (Dh = 59 nm).

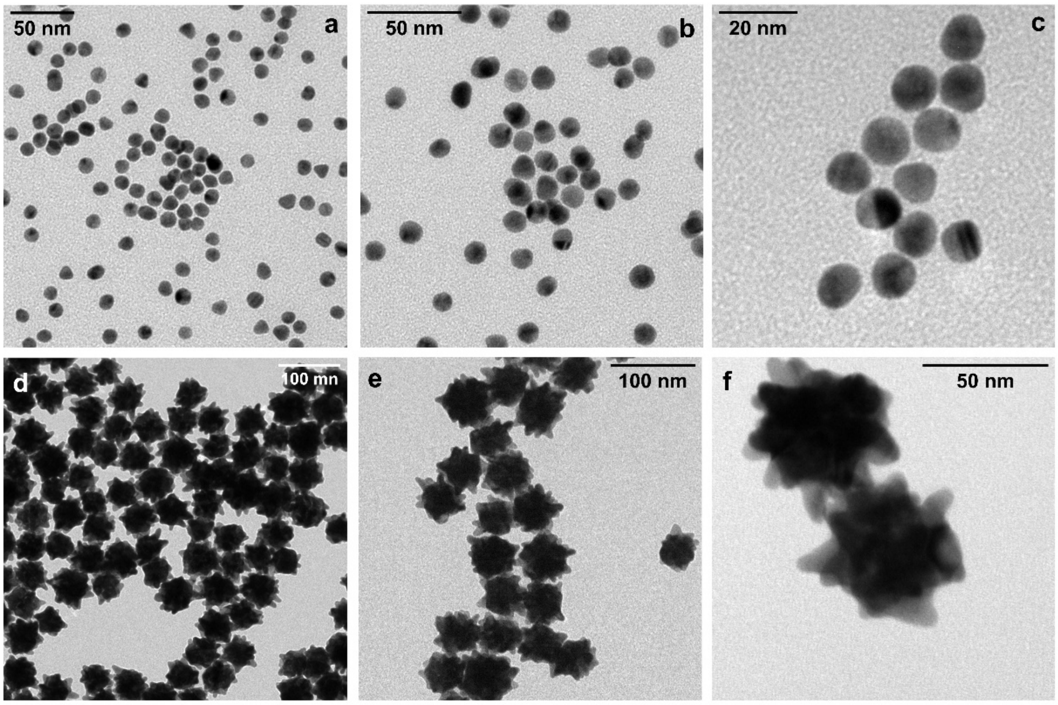

Figure S2. Transmission Electron Microscopy (TEM) analysis. (**a**-**c**) Representative images of citrate-capped gold nanoparticles (GNPs) used as seeds. (**d**-**f**) Representative images of multibranched gold nanoparticles (MGNPs).

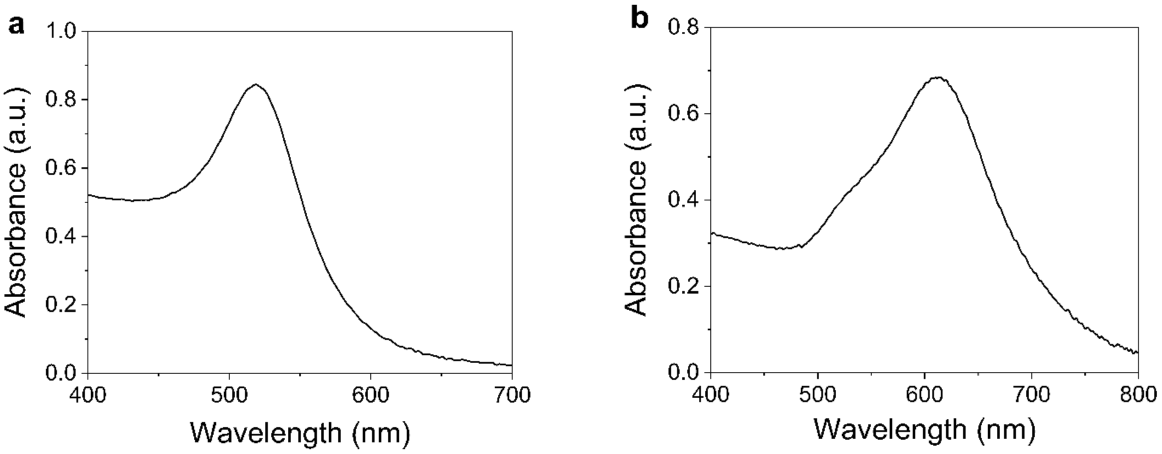

Figure S3. UV-visible spectroscopy analysis. (**a**) UV-vis absorption spectra in water of citrate-capped gold nanoparticles (GNPs) used as seeds. (**b**) UV-vis absorption spectra in water of multi-branched gold nanoparticles (MGNPs).

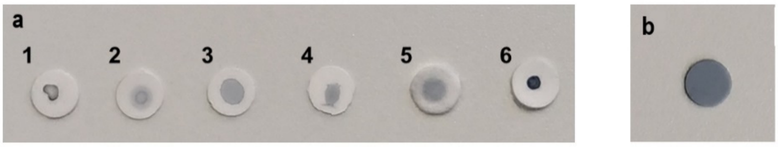

Figure S4. Substrate material screening and deposition optimization. (**a**) Circle pieces (6 mm Ø) of several substrates (1- polyvinylidene difluoride, 2- nitrocellulose membrane, 3- cellulose nitrate membrane, 4- cellulose acetate membrane, 5- cellulose filter paper and 6- polyamide membranes) were drop casted with 0.4 μL of MGNPs (61 pM, λ_{max} = 647 nm, absorbance = 12, $\varepsilon = 1.96 \times 10^{10}$ M^{-}

[1]cm^{-1}) and subsequently dried in a vacuum chamber. (**b**) Circle piece (6 mm Ø) of polyamide membrane functionalized with MGNPs (28.5 pM, λ_{max} = 647 nm, absorbance = 0.9, ε = 31.13 × 10^9 $M^{-1}cm^{-1}$, in HEPES buffer 25 mM, pH = 7.0) using an optimized deposition technique. This method relies on decreasing the concentration of the MGNPs and use a Kitasato becker connected to a vacuum pump to achieve a more uniform particle deposition.

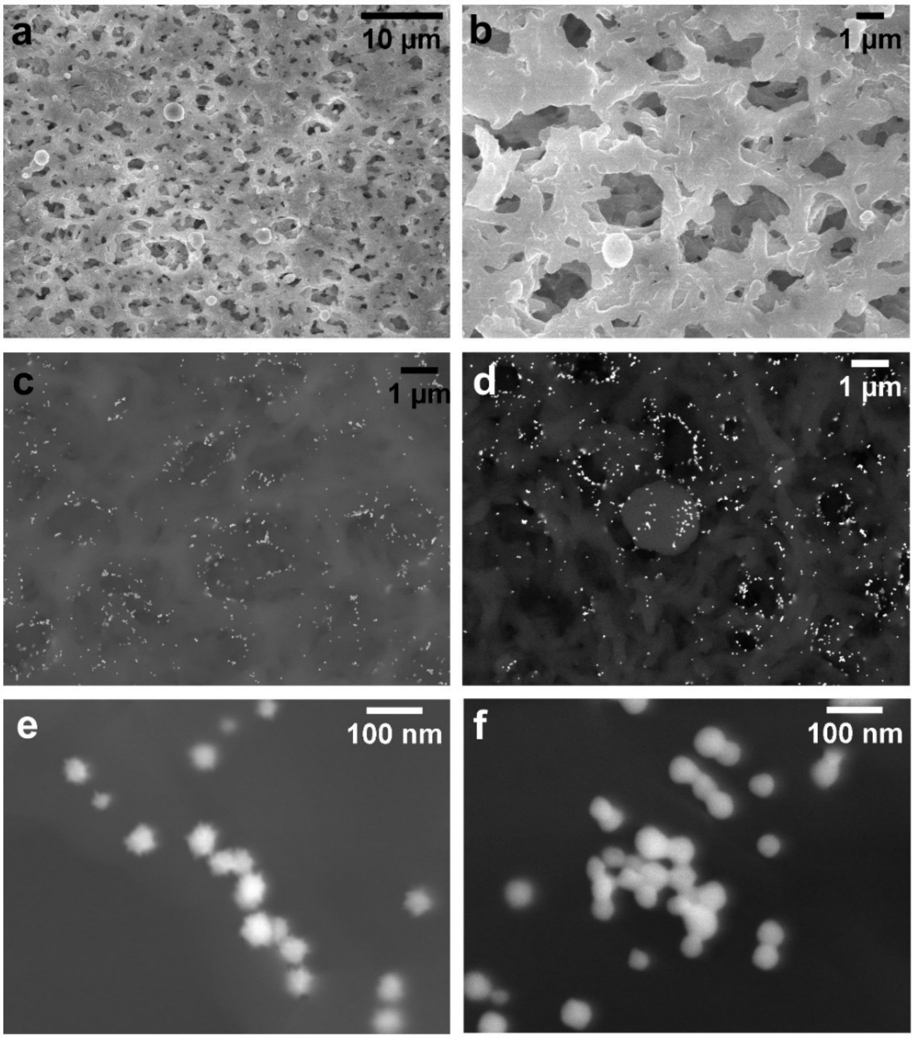

Figure S5. Scanning electron microscopy (SEM) analysis. (**a**, **b**) Polyamide membrane (pore size 0.2 μm). (**c**, **e**) MGNPs on polyamide membranes pre-test. (**d**, **f**) Spherical GNPs on polyamide membranes after testing glucose spiked saliva.

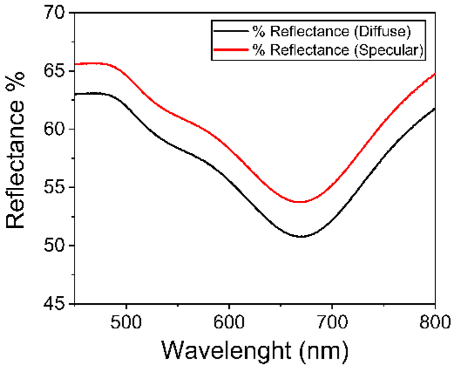

Figure S6. Reflectance spectra analysis. Reflectance spectroscopy plots in diffuse and specular modes of a healthy donor saliva sample (1.5 mg/dL) at an initial time. Specular and diffuse reflectance spectra are very similar. It means that the polyamide membrane functionalized with MGNPs and GOx, wet with the saliva sample and then covered with an optical plastic, reflects light intensity almost homogeneously in all directions. The homogeneous light reflection is a typical phenomenon of isotropic materials. Even though, results in specular (red) or diffuse (black) mode show small differences, we chose diffuse mode to perform the measures for its widespread use in pigment characterization[4].

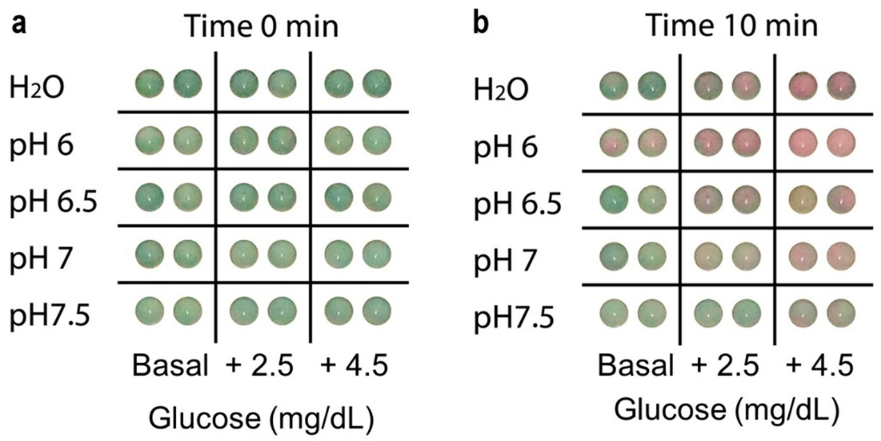

Figure S7. Buffering pH condition optimization. Pictures of one donor experimental evaluation of pH on the glucose detection assay. (**a**) Assay at the initial time 0 minutes. (**b**) Assay at the final time 10 minutes. Functionalized polyamide membranes loaded with saliva samples mixed with the NaI buffered solution in a 2:1 ratio (buffer conditions: double distilled water (non-buffered) and sodium phosphate buffer (300 mM) at four pH: 6, 6.5, 7 and 7.5). Saliva samples were previously spiked 2.5 and 4.5 mg/dL with D-(+)-Glucose to simulate concentrations of 4, and 6 mg/dL, considering a basal level of glucose of 1.5 mg/dL.

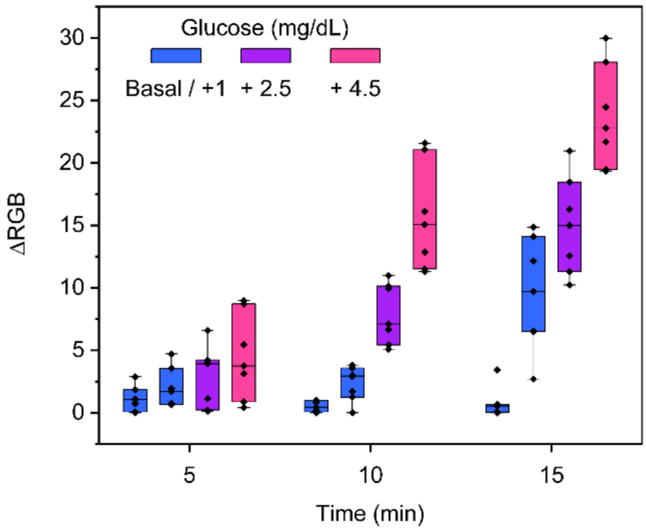

Figure S8. Device performance dependence from time. Device performance assessment in seven saliva samples from healthy donors. Considering a basal glucose concentration of 1.5 mg/dL, samples were spiked with 1, 2.5 and 4.5 to simulated glucose levels of 2.5, 4, and 6 mg/dL. The RGB coordinates values were measured from pictures at t = 0, t = 5, t = 10, and t = 15 min with ImageJ.

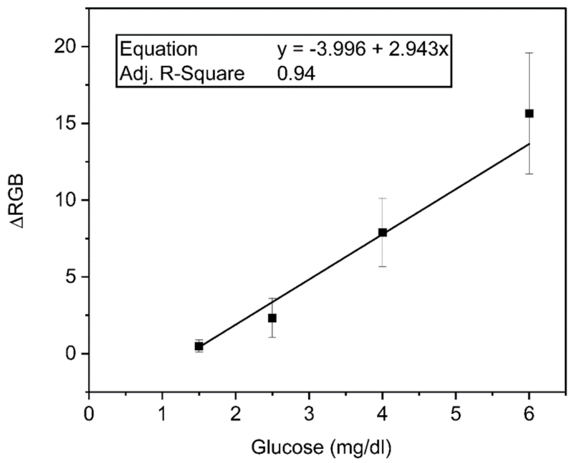

Figure S9. Analytical curve used to assess LOD and LOQ of the device. The plot displays the analytical curve and its linear regression equation y = −3.996 + 2.943 × x with a R^2 of 0.94. LOD and LOQ are 2 and 3.2 mg/dL respectively. These parameters were calculated using Equation (1) and (2), and subsequently using the lineal regression equation.

$$Y_{LOD} = y_{(blank)} + 3.3 * SD_{(blank)} \quad (1)$$
$$Y_{LOQ} = y_{(blank)} + 10.1 * SD_{(blank)} \quad (2)$$

References

1. Turkevich, J., Stevenson, P. C. & Hillier, J. A study of the nucleation and growth processes in the synthesis of colloidal gold. *Discussions of the Faraday Society* **1951**, *11*.
2. FRENS, G. Controlled Nucleation for the Regulation of the Particle Size in Monodisperse Gold Suspensions. *Nat. Phys. Sci.* **1973**, *241*, DOI:10.1038/physci241020a0.
3. Maiorano, G. et al. Monodispersed and size-controlled multibranched gold nanoparticles with nanoscale tuning of surface morphology. *Nanoscale* **2011**, *3*, *2227-32;* DOI:10.1039/c1nr10107b.
4. Yivlialin, Galli, Raimondo, Martini & Sassella. Detecting the NIR Fingerprint of Colors: The Characteristic Response of Modern Blue Pigments. *Heritage* **2019**, *2*, 2255-2261*;* DOI:10.3390/heritage2030137.
